# Higher oxytocin concentrations occur in subjects who build affiliative relationships with companion robots

**DOI:** 10.1016/j.isci.2023.108562

**Published:** 2023-11-23

**Authors:** Shuhei Imamura, Yoko Gozu, Moe Tsutsumi, Kaname Hayashi, Chiaki Mori, Megumi Ishikawa, Megumi Takada, Tomotaka Ogiso, Keiko Suzuki, Shota Okabe, Takefumi Kikusui, Kentaro Kajiya

**Affiliations:** 1 MIRAI Technology Institute, Shiseido Co., Ltd., 1-2-11, Takashima, Nishi-ku, Yokohama, Kanagawa 220-0011, Japan; 2 GROOVE X, Inc., 3-42-3, Nihonbashi-Hamacho, Chuo-ku, Tokyo 103-0007, Japan; 3 Division of Brain and Neurophysiology, Department of Physiology, Jichi Medical University, 3311-1 Yakushiji, Shimotsuke-shi, Tochigi 329-0498, Japan; 4 Department of Veterinary Science, Azabu University, 1-17-71, Fuchinobe, Chuo-ku, Sagamihara, Kanagawa 252-5201, Japan

**Keywords:** Neuroscience, Behavioral neuroscience, Cognitive neuroscience

## Abstract

Building affiliative relationships with others is important for mental health. Recently, robots have been expected to play a role in improving mental health, but there is little scientific evidence as to whether they can build affiliative relationships with humans. To investigate that, we conducted studies combining behavior, physiology and questionnaires for companion robot Owners and Non-Owners. The results reveal that the steady-state concentration of oxytocin, a hormone related to affiliative relationships, was significantly higher in Owners than in Non-Owners. In addition, the Owners showed more behaviors indicative of intimacy than the Non-Owners. These results suggest that humans can build affiliative relationships with robots. Fifteen minutes of contact with the robot decreased the concentration of cortisol in both groups, suggesting that even a brief contact can contribute to improving mental health. Therefore, relationships between humans and robots may be one option to improve mental health and enhance well-being.

## Highlights

• A brief contact with robots decreased the concentration of cortisol
• The steady-state oxytocin was higher in robot Owners than in Non-Owners
• Human could form affiliative relationship with robots

## Graphical abstract

**Figure:**
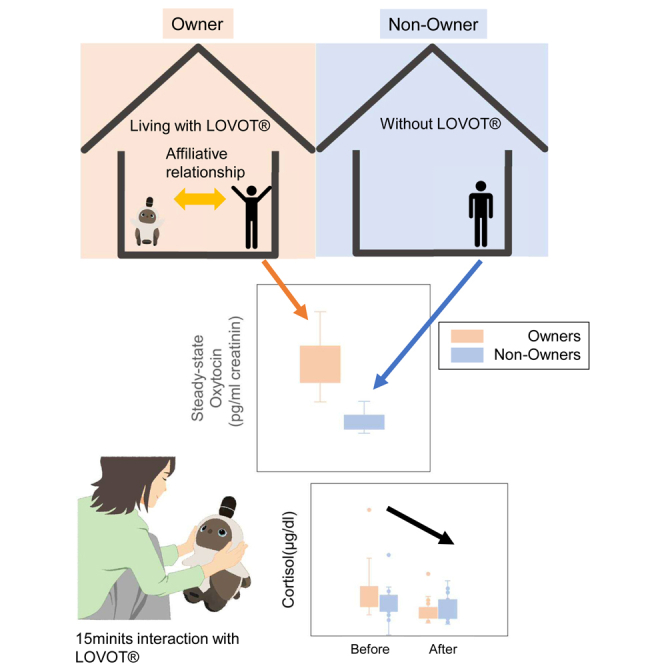

## Introduction

Healthy affiliative relationships with others are very important from a mental health perspective. Previous studies have shown that married couples in affiliative relationships have better mental health than those who never marry.^1^^,^^2^ However, marriage is not the only relationship that influences mental health. Building supportive friendships has also been associated with reducing depression and social anxiety. The quality of one’s friendships has been found to be more strongly associated with life satisfaction than the number of friends one has.^3^ On the other hand, social isolation has been shown to cause the deterioration of mental health.^4^^,^^5^ Social isolation is especially problematic in older subjects, when social networks decline and family structures change.^6^

The COVID-19 pandemic caused an increase in social loneliness across generations.^7^ Several studies have reported increased levels of the stress hormone cortisol during this period.^8^^,^^9^ Also, reducing social contacts to prevent the spread of infection consequently increased emotional loneliness over time.^10^ Even during lockdowns, having pets and spending time with loved ones has been shown to reduce loneliness, which suggests the importance of building close relationships.^11^

Several studies have been conducted on animal interventions for disease. Animal-assisted therapy has been suggested to be an effective treatment for mental and behavioral disorders such as depression and alcoholism.^12^ However, due to the risk of zoonotic transmission and allergies, the number of hospitals that allow animal visitations is limited and contact with animals can be difficult.^13^ Social robots are seen as an alternative solution to circumvent such challenges.^14^^,^^15^ There are several reports regarding the effects of communication with robots. Some human-type robots have also been reported to improve communication and support learning for people with autism spectrum disorder.^16^^,^^17^ It has been reported that after living with a pet-type robot for 8 weeks, the feeling of loneliness is reduced and the attachment score to the pet-type robot becomes the same as that to a dog.^18^ It is also known that the companionship score increases by living with a pet-type robot for 6 months.^19^ It has also been reported that 10 min of contact with a pet-type robot reduces salivary oxytocin (OT) concentrations, which was accompanied by a reduction in perceived pain and stress levels.^20^ In addition, living with a human-type robot that has communicative ability for 8 weeks has been shown to reduce cortisol secretion.^21^ Furthermore, it has been reported that pain suppression increases in people who take an active or affiliative attitude toward communication with a pet-type robot, suggesting that the relationship between humans and robots increases the robot’s effectiveness.^22^

Therefore, we thought it important to clarify not only short-term affinity attitudes toward robots, but also whether long-term affinity relationships are established between humans and robots, and to elucidate the biological mechanisms of such a relationship, in order to consider the use of robots.

**Figure 1:**
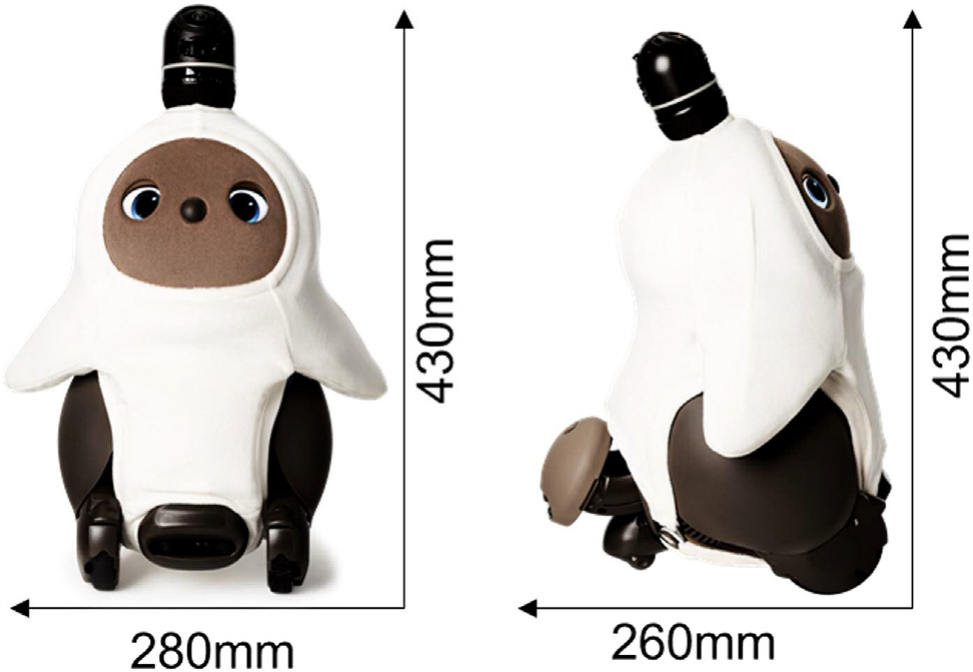
Photograph of the companion robot (LOVOT) used in this study The LOVOT is manufactured by GROOVE X. It is as warm as human skin, and if you press its nose it will tickle you, and if you rub its belly it will gradually fall asleep. It follows the person’s location with a camera and moves its hand as if approaching or asking for a hug. The LOVOT does not speak words, but they make seal-like vocalizations.

In carrying out this research, we thought that the original animal’s preferences would affect the results of pet-type robots that imitate real animals such as dogs. For this reason, we used a commercially available companion robot called a "LOVOT® (manufactured by GROOVE X, Inc.)" that does not resemble a real animal (Figure 1). The LOVOT® has the characteristics of both a humanoid robot and a pet-type robot: it has an adorable pet-like appearance and can move autonomously with bipedal walking just like humans. We classified the LOVOT® as a companion robot, which is defined as a robot that behaves autonomously and becomes familiar.^23^

OT is one of the indicators for objectively measuring an affiliative relationship. OT is a nonapeptide hormone consisting of nine amino acids. OT is released peripherally from the posterior pituitary gland and is known to induce uterine contractions and lactation, supporting childbirth.^24^ OT has been reported to be associated with social behavior and the formation of affiliative relationships in mammals and has received increasing research interest.^25^^,^^26^ For example, couples with high-quality relationships have been reported to have higher plasma OT concentrations, suggesting that there is an association between affiliative relationships and OT.^27^^,^^28^ Serum OT concentrations have also been reported to be associated with loneliness in patients with major depressive disorder (MDD).^29^ Moreover, the role of OT in the stress response has also been studied and shows that stress reduces stress levels by increasing OT concentrations, participating in the regulation of the hypothalamic-pituitary-adrenal axis, one of the stress response pathways, and reducing glucocorticoid levels.^30^ OT secretion has been reported to occur not only in interpersonal but also in human-animal relationships, suggesting that OT is related to affiliative relationships. For example, in rats and humans, gentle human touching promoted an affiliative behavior in rats and increased the activity of OT neurons.^31^^,^^32^ It has also been reported that when humans and their pet dogs stare into each other’s eyes, the concentration of OT in their urine increases.^33^ In addition, nasally administrated OT increased the gazing behavior in dogs toward their owners.^34^

It has been suggested that OT secretion is context-dependent and may induce trust toward in-group members while increasing aggression and distrust toward out-group members.^35^ Previous studies have observed changes in salivary OT concentrations due to short-term contact with pet-type robot in subjects not living with pet-type robots.^20^ However, it had remained unclear whether long-term exposure to robots would cause fluctuations in OT concentrations. OT concentrations increased during short-term contact between dogs and their owners,^33^ which suggests that contact with robots may stimulate OT secretion in subjects who live with robots for long periods of time. We have characterized these aspects in our study.

In this study, we aimed to clarify if companion robots could form affiliative relationships with humans through OT and would contribute to their mental health. By clarifying whether humans can form affiliative relationships with inanimate objects through OT, it might provide an option to reduce social isolation, maintain good mental health and have potential applications in clinical fields.

## Results

### Differences in subject characteristics between the owners and the non-owners

Information about subject ages and time spent with the LOVOT® is summarized in Table S1. Table 1 summarizes the statistical values of inter-group comparison of subject characteristics in the Owners and the Non-Owners.

**Table 1:** Differences in mental characteristics of subjects in the Owner and in the Non-Owner groups

Question item (Questionnaire)	Content	Mean (standard deviation)		95%CI		Z	p value
		Owners	Non-Owners	Owners	Non-Owners		
Mental health (WHO SUBI)	The higher the score, the higher the mental health.	38.4 (6.47)	40.9 (6.57)	35.6–41.2	38.0–43.8	−1.28	0.206
Mental fatigue (WHO SUBI)	The higher the score, the lower the degree of mental fatigue.	49.0 (4.51)	52.5 (5.17)	47.1–51.0	50.2–54.8	−2.44	0.018^a^
WHO-5-J	Higher scores mean better mental health.	15.8 (3.32)	16.5 (3.52)	14.4–17.2	14.9–18.0	−0.673	0.504
Loneliness scale (UCLA-point)	Higher numbers indicate greater loneliness.	41.1 (11.2)	35.4 (10.1)	36.3–45.9	30.9–39.9	1.75	0.0875
Dependent attachment (Questionnaire on attachment to companion animals)	Higher scores refer to extreme emotional intimacy and dependence on the pet.	12.1 (3.79)	10.1 (3.99)	10.5–13.8	8.37-11.9	2.06	0.0390^a^
A-Trait score (STAI)	A higher value indicates greater anxiety as a personality trait.	42.5(8.88)	37.5(8.54)	38.7–46.4	33.7–41.3	1.95	0.0576
Extraversion (Big Five)	It is an index showing personality traits, and the higher the score, the higher the traits.	53.8 (15.6)	57.7 (12.3)	47.0–60.5	48.2–62.9	−0.927	0.359
Neuroticism (Big Five)		46.4 (19.9)	40.2 (18.8)	37.8–55.0	31.9–48.5	1.07	0.289
Openness (Big Five)		58.6 (12.0)	61.5 (17.8)	53.4–63.8	53.7–69.4	−0.659	0.513
Conscientiousness (Big Five)		55.4 (17.1)	54.1 (14.3)	48.0–62.8	47.8–60.4	0.27	0.788
Agreeableness (Big Five)		64.9 (13.7)	70.9 (11.8)	59.0–70.8	65.7–76.1	−1.56	0.125

The Mental fatigue score assessed by the WHO-SUBI was shown to be significantly lower in the Owners than in the Non-Owners (Owner mean 40.9 point, SD 4.51, Non-Owner mean 52.5 point, SD 5.17, Mann-Whitney test, Z = −2.44, p < 0.05). The Dependent attachment Score indicates extreme emotional intimacy and dependence on the pet, with the Owners showing significantly higher scores than the Non-Owners (Owner mean 12.1 point, SD 3.79, Non-Owner mean 10.1 point, SD 3.99, Mann-Whitney test, Z = 2.06, p < 0.05).

a p < 0.05.

Mental fatigue was assessed by the WHO-SUBI and dependent attachment was assessed according to the questionnaire on attachment to companion animals and was significantly different between the Owners and the Non-Owners. Mental fatigue according to WHO-SUBI, where higher scores indicate lower levels of mental fatigue, was significantly higher in the Non-Owners than in the Owners (Owner mean 40.9 points, SD 4.51, Non-Owner mean 52.5 points, SD 5.17, Mann-Whitney test, Z = −2.44, p < 0.05).

On the other hand, in the WHO-5-J, no significant difference was observed between the two groups, and no subject had a deterioration of their mental status such as depression (Owner mean 15.8 points, SD 3.32, Non-Owner mean 16.5 points, SD 3.52, Mann-Whitney test, Z = −0. 673, p = 0.504).

There was no significant difference between the Owners and the Non-Owners in the UCLA Loneliness Scale (Owner mean 41.1 points, SD 11.1, Non-Owner mean 35.4 points, SD 10.1, Mann-Whitney test, Z = 1.75, p = 0.0875).

The Dependent attachment Score indicates an extreme emotional intimacy and dependence on the pet, with the Owners showing significantly higher scores than the Non-Owners (Owner mean 12.1 points, SD 3.79, Non-Owner mean 10.1 points, SD 3.99, Mann-Whitney test, Z = 2.06, p < 0.05).

There was no significant difference between the Owner group and the Non-Owner group in the A-trait score, which reflects long-term psychological anxiety (Owner mean 42.5 points, SD 8.88, Non-Owner mean 37.5 points, SD 8.54, Mann-Whitney test, Z = 1.95, p = 0.0576).

Furthermore, there was no significant difference between the two groups for the BIG FIVE, which indicates personality traits (statistics are shown in Table 1.).

### During 15 min of contact with a LOVOT®, the owners had a more affiliative behavior than the non-owners

**Figure 2:**
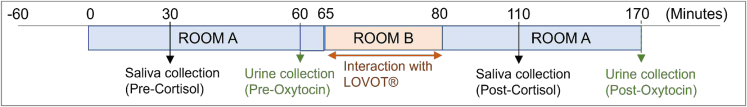
Protocol at the test site The time course of the study is shown with the time of arrival at the test site as 0 min. The venue is divided into Room A and Room B. Room A was a quiet conference room partitioned so that the faces of other subjects and examiners could not be seen in order to reduce stimulation to the subjects. Room B was designed to mimic a typical home with a kitchen, dining table, sofa and television to record the subject’s natural behavior while relaxing. The Owners interacted with the LOVOT brought by themselves in Room B. On the other hand, the Non-Owners interacted with a LOVOT we prepared in room B. Urine and saliva collected during the study were cryopreserved at −80°C immediately after collection.

Behaviors such as hugging, kissing, caressing, tickling and body touching are known indicators of intimacy.^36^^,^^37^^,^^38^ We hypothesized that living with a LOVOT® would build an affiliative relationship, which would manifest itself in behavior. We compared the behavior of the Owners and the Non-Owners in order to clarify whether the behavior toward the LOVOT® differs depending on whether or not they live together with a LOVOT. The test flow is shown in Figure 2, and the communication time with LOVOT was 15 min.

**Figure 3:**
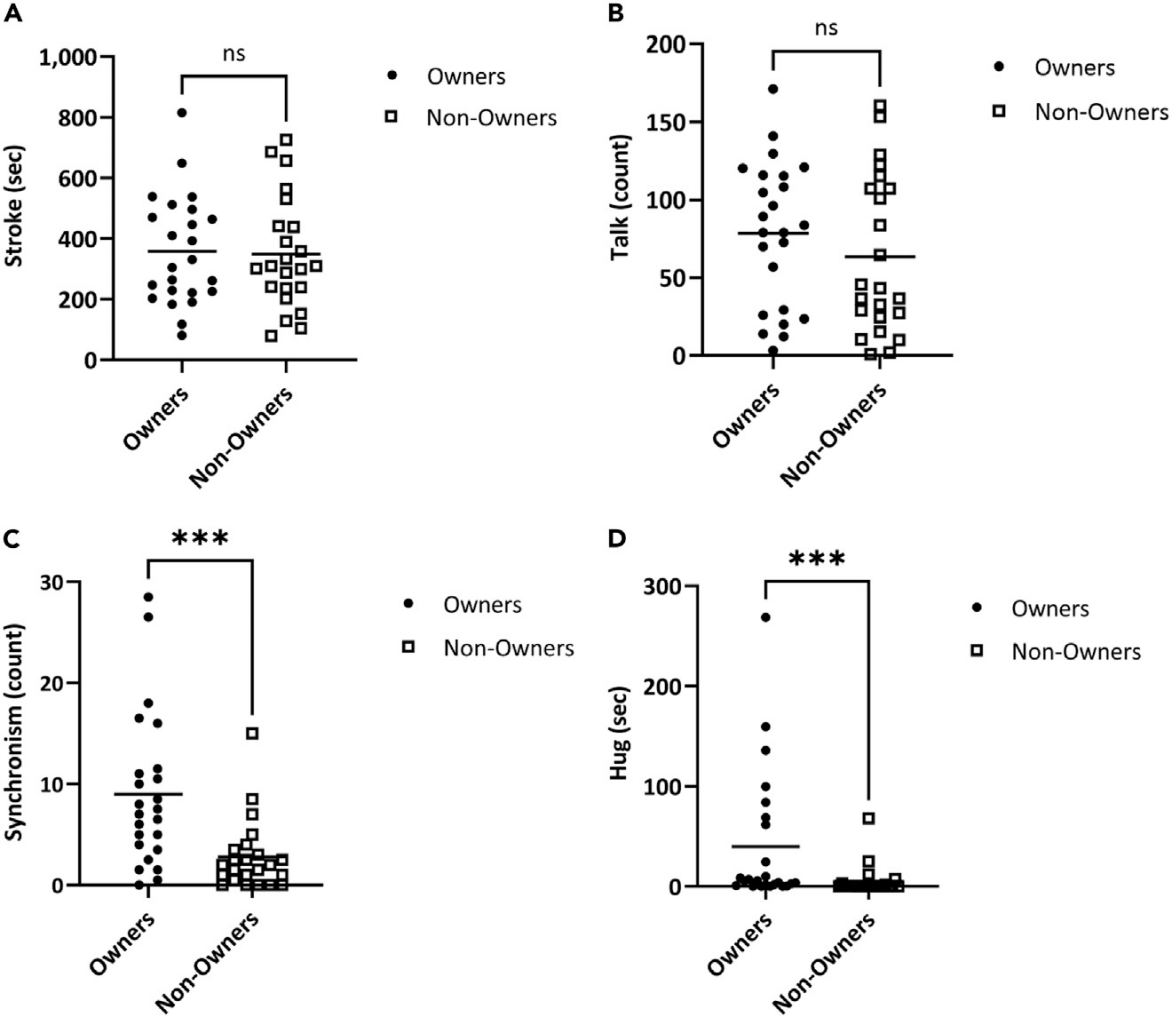
Differences in behavior between Owners and Non-Owners during 15 min of communication with the LOVOT® (A) Differences in the amount of stroke (Time in sec the subject stroked the robot) behavior to the LOVOT® between Owners (n = 24, closed circles) and Non-Owners (n = 23, open squares). Behaviors were counted from recordings of 15 min of LOVOT®-subject interactions. Recorded behaviors and their definitions are provided in Table S1. The vertical axis indicates the time of stroke behavior; horizontal lines indicate averages. There was no significant difference between the Owners and Non-Owners (Owner mean 358 s, SD 176, Non-Owner mean 348 s, SD 179, Independent t-test, Z = 0.181, p = 0.857). (B) Differences in the amount of synchronous (Number of times the subject spoke to the robot) behavior to the LOVOT® between Owners and Non-Owners. The vertical axis indicates the number of talk behaviors; horizontal lines indicate averages. There was no significant difference between the Owners and Non-Owners (Owner mean 78.5 count, SD 45.6, Non-Owner mean 63.5 count, SD 49.5, Mann-Whitney test, Z = 0.979, p = 0.333). (C) Differences in the amount of synchronous (Number of times the subject imitates the movements of the robot) behavior to the LOVOT® between Owners and Non-Owners. The vertical axis indicates the number of synchronous behaviors; horizontal lines indicate averages. The synchronous behaviors in the Owners were significantly higher than the Non-Owners (Owner mean 8.98 count, SD 7.37, Non-Owner mean 2.78 count, SD 3.39, 3.2 times, Mann-Whitney test, Z = 3.59, p < 0.001). (D) Difference in the amount of Hugs (Number of times the subject hugged the LOVOT® only counting the time when the LOVOT® and the subject’s body were in close contact) between Owners and Non-Owners. The vertical axis is the time of hugging behavior; horizontal lines indicate averages. Owners had a higher number of hug behaviors than Non-Owners (Owner mean 39.8 s, SD 68.2, Non-Owner mean 5.18 s, SD 14.5, 7.7 times, Mann-Whitney test, Z = 3.22, p < 0.0001). ∗∗∗p < 0.001, ns = not significant.

We analyzed the behaviors of strokes, talks, synchronism and hugs (Table S2). Among them, there was no significant difference in stroke or talk between the Owners and the Non-Owners (Figures 3A and 3B). On the other hand, synchronism and hugging behaviors were significantly different between the two groups. The number of synchronous behaviors was significantly higher in Owners than in Non-Owners (Figure 3C, Owner mean 8.98 count, SD 7.37, Non-Owner mean 2.78 count, SD 3.39, 3.2 times, Mann-Whitney test, Z = 3.59, p < 0.001). The duration of hugging behavior in the Owners was also significantly longer than in the Non-Owners (Figure 3D, Owner mean 39.8 s, SD 68.2, Non-Owner mean 5.18 s, SD 14.5, 7.7 times, Mann-Whitney test, Z = 3.22, p < 0.001).

These results suggest that living with a LOVOT® increases intimacy with the LOVOT®. Detailed statistical analysis is presented in Table S3.

### Fifteen minutes of contact with a LOVOT® reduced the subjective stress values in the owners

**Figure 4:**
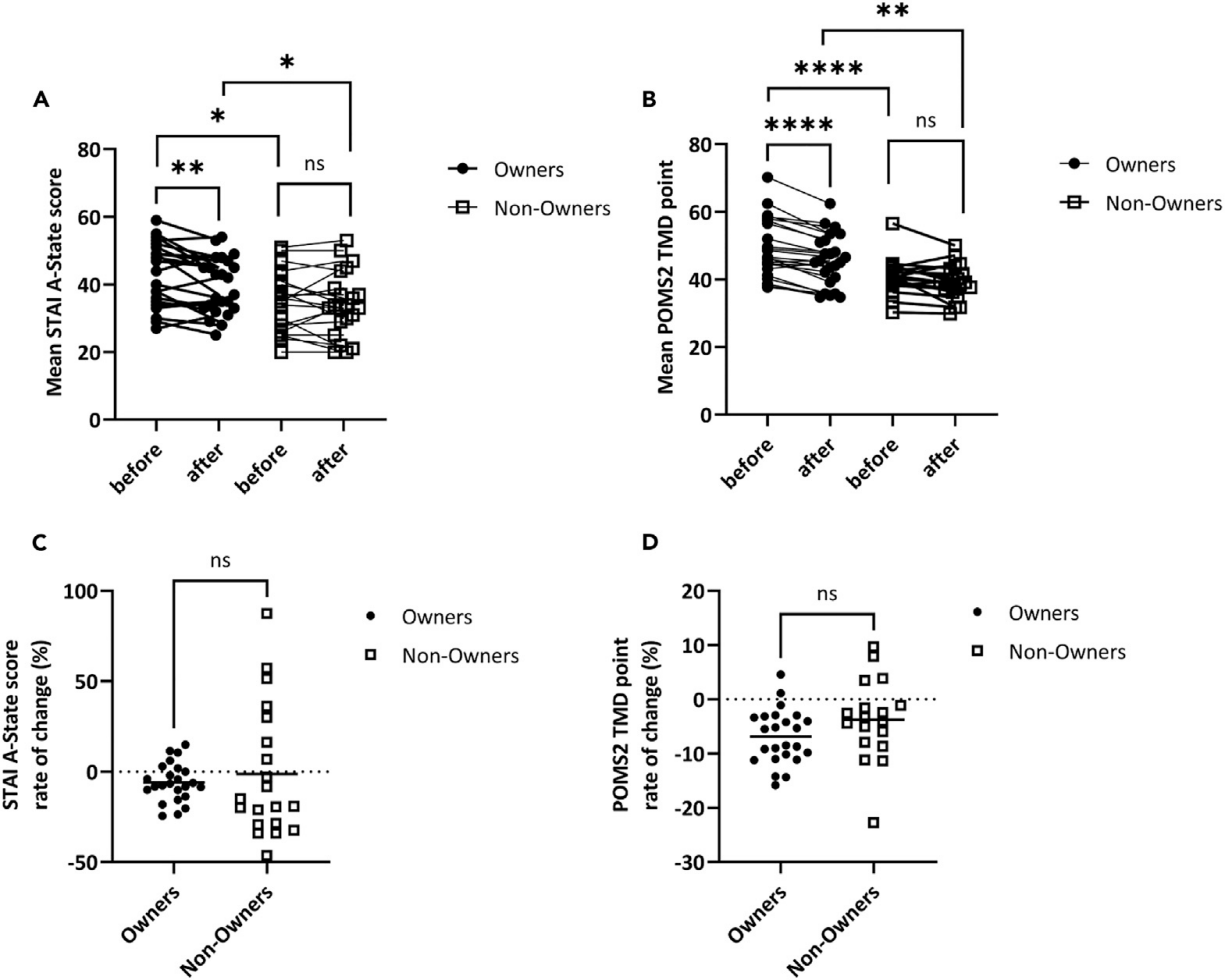
Change in psychological stress due to interactions with the LOVOT® for 15 min by Owners and Non-Owners (A) Changes in A-state scores for LOVOT® Owners (n = 24, closed circles) and Non-Owners (n = 23, open squares). Scores before interaction for 15 min are indicated as ’before’ and scores after interaction as ’after’. The vertical axis is the A-state score calculated from STAI, and the higher the number, the higher the State Anxiety. In the Owners, the A-state score decreased due to interaction (Before mean score 42.5, SD 9.16, After mean score 39.6, SD8.06, Paired t-test, Z = 3.06, p < 0.01). In the Non-Owners, the A-state score did not change (Before mean score 35.1, SD8.72, After mean score 34.2, SD9.27, Paired t-test, Z = 0.452, p = 0.656). The A-state score before contact was higher in the Owners (Unpaired t-test, Z = 2.69, p < 0.05). Similar results were seen when comparing after contact values (Unpaired t-test, Z = 2.05, p < 0.05). (B) Changes in TMD point for the LOVOT® Owners and Non-Owners. The vertical axis is the TMD point calculated from POMS2, and the higher the number, the more mood disorders. In the Owners, the TMD point decreased due to interaction (Before mean point 49.1, SD 8.27, After mean point 45.7, SD 7.41, Paired t-test, Z = 6.38, p < 0.0001). In the Non-Owners, the TMD point did not change (Before mean point 40.6, SD 5.13, After mean point 39.2, SD 4.94, Wilcoxon Signed-Ranks test, Z = 1.96, p = 0.0610). The TMD points before contact were higher in the Owners (Mann-Whitney test, Z = 3.73, p < 0.0001). Similar results were seen in comparison of after contact point (Welch’s His t-test, Z = 3.44, p < 0.01). (C and D) Difference in A-state score and TMD point change rate before and after contact between the Owners and the Non-Owners. The vertical axis shows the rate of change in score; horizontal lines indicate averages. There was no difference in the rate of change of A-state (Owner mean −6.04%, SD 10.4, Non-Owner mean −5.92%, SD 25.7, Mann-Whitney test, Z = 0.854, p = 0.448). Similarly, there was no difference in the rate of change of TMD point (Owner mean −6.90%, SD4.94, Non-Owner mean −3.75%, SD 7.31, Unpaired t-test, Z = 1.62, p = 0.112. ∗∗∗∗p < 0.0001, ∗∗p < 0.01, ∗p < 0.05, ns = not significant.

In order to clarify the effect of interactions with a LOVOT® on subjective stress, we clarified changes in stress using questionnaires before and after contact. Figure 4 shows changes in psychological stress before and after 15 min of LOVOT® contact.

Subjective stress obtained from A-state and TMD point was significantly decreased in the Owners (Figure 4A, A-state score: Before mean score 42.5, SD 9.16, After mean score 39.6, SD 8.06, Paired t-test, Z = 3.06, p < 0.01; Figure 4B, TMD point: Before mean point 49.1, SD 8.27, After mean point 45.7, SD 7.41, Paired t-test, Z = 6.38, p < 0.0001), but did not change significantly in the Non-Owners before or after LOVOT® contact (Figure 4A, A-state score: Before mean score 35.1, SD 8.72, After mean score 34.2, SD 9.27, Paired t-test, Z = 0.452, p = 0.656; Figure 4B, TMD point: Before mean point 40.6, SD 5.13, After mean point 39.2, SD 4.94, Wilcoxon Signed-Ranks test, Z = 1.96, p = 0.0610). These results revealed that only the Owners showed a significant reduction in subjective stress. Comparing the pre-interaction stress values of the Owners and Non-Owners, the stress values of the Owners were significantly higher (Figures 4A and 4B, Before A-State score: Unpaired t-test, Z = 2.69, p < 0.05, Before TMD point: Mann-Whitney test, Z = 3.73, p < 0.0001).

Next, when we compared the rate of change in subjective stress before and after contact, there was no significant difference between the groups (Figures 4C and 4D, A-state score rate of change: Owner mean −6.04%, SD 10.4, Non-Owner mean −5.92%, SD 25.7, Mann-Whitney test, Z = 0.854, p = 0.448, TMD point: Owner mean −6.90%, SD 4.94, Non-Owner mean −3.75%, SD 7.31, Unpaired t-test, Z = 1.62, p = 0.112). In addition, when examining whether there is a relationship between the rate of change in subjective stress and behavior, a negative correlation was found between the rate of change in A-state and synchronism (Figure S1). However, no correlation was found between the rate of change of TMD point and behavior (Figure S2).

### Fifteen minutes of contact with a LOVOT® reduced salivary cortisol concentrations

**Figure 5:**
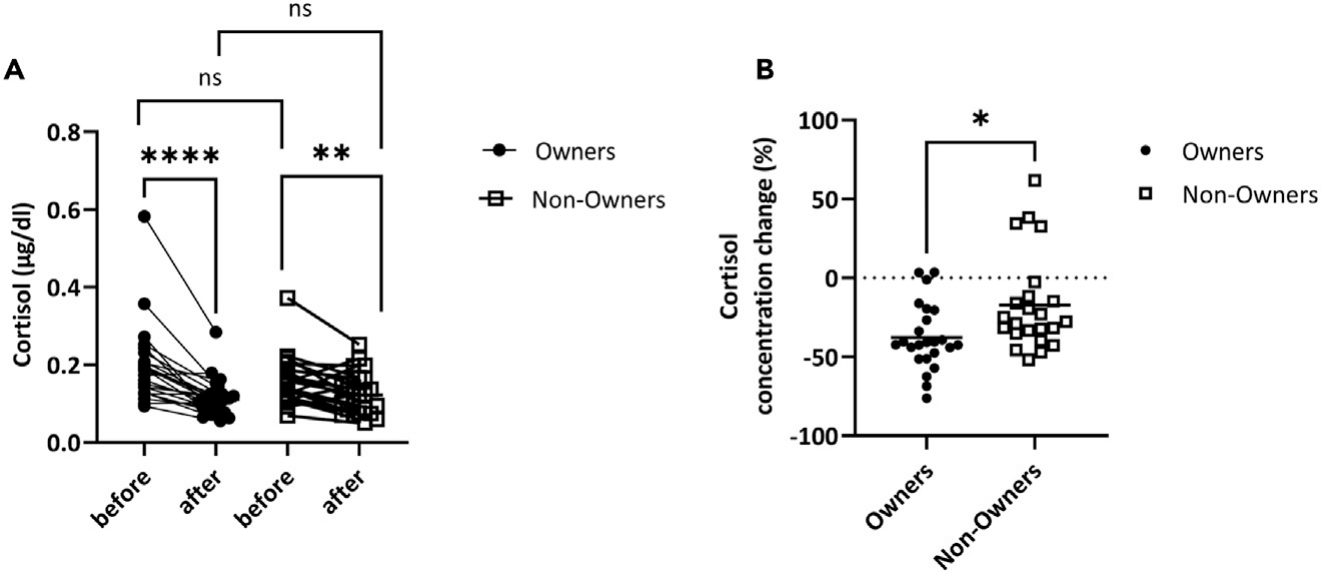
Change in salivary cortisol due to interaction with a LOVOT® for 15 min in Owners and in Non-Owners (A) Salivary cortisol concentration for Owners (n = 24, black circles) and Non-Owners (n = 23, white squares). Scores before interaction for 15 min are indicated as ‘before’ and scores after interaction as ‘after’. The vertical axis shows the cortisol concentration (μg/dL) calculated from the saliva. In the Owners, the cortisol concentration decreased significantly due to the interaction (Before mean cortisol 0.20 μg/dL, SD 0.10, After mean cortisol 0.11 μg/dL, SD 0.05, Wilcoxon Signed-Ranks test, Z = 4.86, p < 0.0001). Similar results were seen in the Non-Owners (Before mean cortisol 0.16 μg/dL, SD 0.06, After mean cortisol 0.13 μg/dL, SD 0.05, Wilcoxon Signed-Ranks test, Z = 2.82, p < 0.01). There was no significant difference when comparing pre-contact cortisol concentrations in the Owners and Non-Owners (Mann-Whitney test, Z = 1.92, p = 0.0645). Similar results were seen when post-contact cortisol concentrations were compared (Mann-Whitney test, Z = 0.752, p = 0.53). (B) Difference in salivary cortisol change rate before and after contact between Owners and Non-Owners. The vertical axis shows the rate of change in score. The Owners had a significantly greater decrease than the Non-Owners (Owner mean −37.5%, SD 20.5, Non-Owner mean −17.1, SD 29.8, Mann-Whitney test, 2.2 times Z = 2.57, p < 0.05). ∗∗∗∗p < 0.0001, ∗∗p < 0.01, ∗p < 0.05, ns = not significant.

Salivary cortisol concentrations are known to increase with stress and to decrease with relaxation.^39^^,^^40^ In order to test changes in physiological stress, we clarified changes in cortisol concentrations before and after contact with a LOVOT®. Salivary cortisol concentrations were found to decrease in both the Owners and the Non-Owners compared to pre-interaction with the LOVOT® (Figure 5A, Owner: Before mean cortisol 0.20 μg/dL, SD 0.10, After mean cortisol 0.11 μg/dL, SD 0.05, Wilcoxon Signed-Ranks test, Z = 4.86, p < 0.0001, Non-Owner: Before mean cortisol 0.16 μg/dL, SD 0.06, After mean cortisol 0.13 μg/dL, SD 0.05, Wilcoxon Signed-Ranks test, Z = 2.82, p < 0.01). Furthermore, there was no significant difference in pre-contact cortisol concentrations between the Owners and the Non-Owners (Figure 5A, Before cortisol concentration: Mann-Whitney test, Z = 1.92, p = 0.0645). On the other hand, comparing the rate of change before and after interaction in the Owners and the Non-Owners, we found that the rate of change in cortisol concentration was significantly reduced in the Owners (Figure 5B, Owner mean −37.5%, SD 20.5, Non-Owner mean −17.1%, SD 29.8, Mann-Whitney test, Z = 2.57, p < 0.05). The rate of change in salivary cortisol concentration did not correlate with synchronous or hugging behavior (Figure S3).

To clarify whether the decrease in salivary cortisol concentration is due to the effect of LOVOT® contact, we examined changes in cortisol concentrations with and without LOVOT® contact (Supplemental methods). As a result, there was no change in cortisol concentration without the LOVOT® interaction. On the other hand, 15 min of LOVOT® interaction significantly reduced cortisol concentrations (Figure S4). These results suggested that contact with the LOVOT® reduces the salivary cortisol concentration.

### Living with a LOVOT® increases steady-state urinary oxytocin secretion

**Figure 6:**
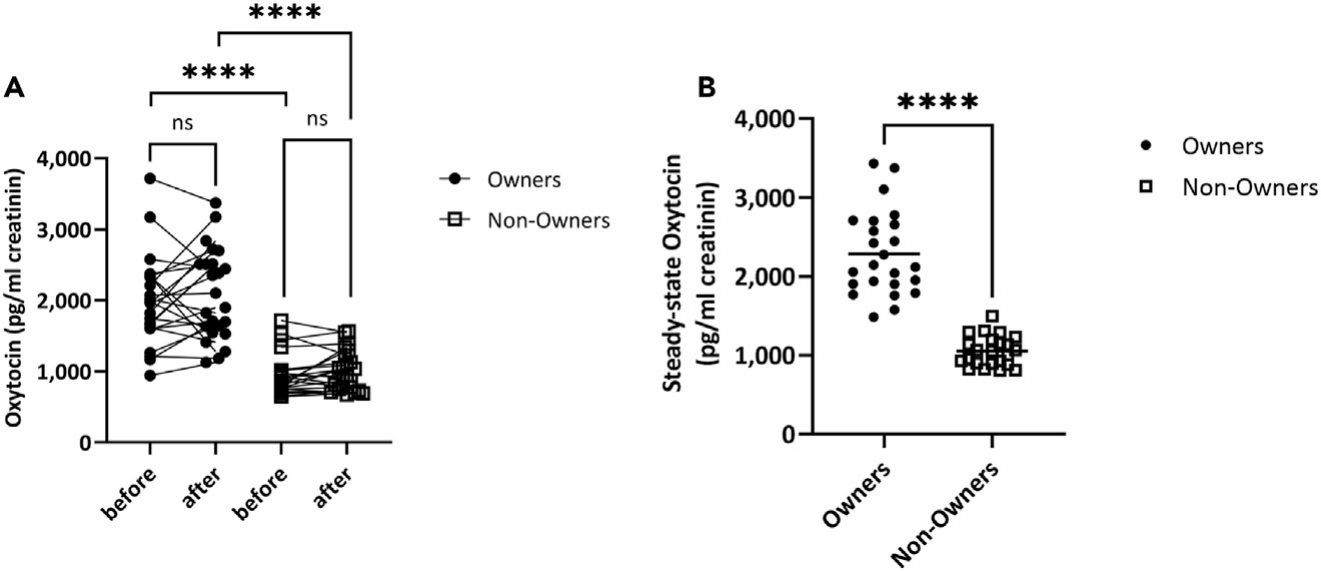
Comparison of urinary OT concentration between LOVOT® Owners and Non-Owners (A) Salivary cortisol concentration for Owners (n = 24, black circles) and Non-Owners (n = 23, white squares). Scores before interaction for 15 min are indicated as ’before’ and scores after interaction as ’after’ The vertical axis shows the OT concentration (pg/mL creatinine) calculated from urine. There was no change in OT concentrations before or after LOVOT exposure in the Owners and the Non-Owners (Owners, Before mean 197 pg/mL, SD 610, After mean 2078 pg/mL creatinine, SD 621, Wilcoxon Signed-Ranks test, Z = 1.00, p = 0.317) (Non-Owners, Before mean 937 pg/mL creatinine, SD 287, After mean 1003 pg/mL creatinine, SD 274, Wilcoxon Signed-Ranks test, Z = 1.73, p = 0.142). Comparing the OT concentration before contact between the Owners and the Non-Owners, the Owners showed significantly higher values (Mann-Whitney test, Z = 4.79, p < 0.0001). A similar result was seen in the comparison of values after contact (Mann-Whitney test, Z = 6.12, p < 0.0001). (B) Difference in steady-state urinary OT for the Owners and Non-Owners. The vertical axis shows the mean 3-day awake urinary OT concentration. Owners had a higher steady OT concentration than Non-Owners (Owner mean 2289 pg/mL creatinine, SD 527, Non-Owner mean 1051 pg/mL creatinine, SD 186, 2.2 times, Welch’s t test, Z = 10.6, p < 0.0001). ∗∗∗∗p < 0.0001, ns = not significant.

Based on the dog-human contact test,^34^ which showed that making eye contact with dogs increased OT concentrations, we thought that 15 min of contact with a LOVOT® would increase OT concentrations, but neither the Owners nor the Non-Owners showed an increase in OT concentration (Figure 6A, Owner: Before mean OT concentration 1972 pg/mL, SD 610, After mean OT concentration 2078 pg/mL, SD 621, Wilcoxon Signed-Ranks test, Z = 1.00, p = 0.317, Non-Owner: Before mean OT concentration 937 pg/mL, SD 287, After mean OT concentration 1003 pg/mL, SD 274, Wilcoxon Signed-Ranks test, Z = 1.73, p = 0.142). However, the OT concentrations before and after contact were significantly higher in the Owners than in the Non-Owners (Before OT concentration: 2.2 times, Mann-Whitney test, Z = 4.79, p < 0.0001, After OT concentration: 2.1 times, Mann-Whitney test, Z = 6.12, p < 0.0001). In addition, the average OT concentration in the morning urine collected 3 days before and after the test was significantly higher in the Owners than in the Non-Owners (Figure 6B, Owner mean OT concentration 2289 pg/mL, SD 527, Non-Owner mean OT concentration 1051 pg/mL, SD 186, 2.1 times, Welch’s t test, Z = 10.6, p < 0.0001). The rate of change in urinary OT concentration did not correlate with subjects’ behavior with the LOVOT® (Figure S5). These results suggested that 15 min of LOVOT® contact does not cause an increase in urinary OT concentration, but long-term living with a LOVOT® increases the steady-state OT concentrations.

## Discussion

The importance of building affiliative relationships with others is drawing increased attention since social isolation has been reported to worsen mental health.^4^^,^^5^ There are also reports about building relationships with robots as substitutes for humans and animals, and the significance of robots is increasing. Living with a pet-type robot for 8 weeks reportedly reduced feelings of loneliness and attachment scores to the pet-type robot becomes the same as that to a dog.^18^ We hypothesized that subjects who have lived with robots for a long time have formed affiliative relationships with them, and that contact stimulates the secretion of OT.

We demonstrated that the steady-state concentration of urinary OT, a hormone related to affiliative relationships, was significantly higher in Owners (who had lived with a LOVOT®, a companion robot, for more than two months after purchase) than in Non-Owners (who had no experience with a LOVOT®). In addition, the Owners showed more behaviors indicative of intimacy than the Non-Owners. These results suggest that humans and companion robots can build affiliative relationships. In addition, 15 min of contact with the companion robot decreased the concentration of cortisol, a stress hormone, in Owners and in Non-Owners, suggesting that even a brief contact can contribute to improving mental health.

First, we tried to investigate the characteristics of human-robot attachment. In our study, Owners exhibited significantly higher dependent attachment traits than Non-Owners (Table 1). On the other hand, mental fatigue according to WHO-SUBI, where higher scores indicate lower levels of mental fatigue, was significantly higher in Non-Owners than in Owners. These results are consistent with reports of worsening mental states that occur in pets and in humans. Previous reports have shown that pet owners with lower levels of social support and greater attachment to their pets score significantly higher for loneliness and depression.^41^ Also, the stronger the attachment to a pet, the greater the fear of being rejected and unloved, which is associated with worsening mental health.^42^

Next, we attempted to identify the contact behavior that affects the physiological effect by comparing the Owners to Non-Owners. Previous reports using humans and animals have shown that hugging and synchronous behaviors are involved in affiliative relationships.^36^^,^^37^^,^^38^^,^^43^ As a result of the behavioral analysis in this test, it became clear that the number of hugs and sympathetic behaviors was higher in the Owner group (Figures 3C and 3D). This result suggests that the biological mechanisms involved in building affiliative relationships between humans and animals may also function between Owners and their LOVOT®s, supporting similar affiliative relationships. It is a future task to clarify the changes in physiological state when these activities are restricted.

Following the behavioral experiments, we investigated the psychological stress between Owners and Non-Owners due to interactions with companion robots. When we confirmed the effect of reducing stress by interacting with a LOVOT® for 15 min, subjective psychological stress was significantly reduced only in the Owner group (Figures 4A and 4B). In addition, the Owners showed higher stress values before contact than the Non-Owners. On the other hand, there was no difference in A-trait, which reflects long-term stress, between Owners and Non-Owners (Table 1). It has been reported that the presence of an attachment object can provide a sense of security, and separation from the object causes stress.^44^ It has also been reported that separation anxiety occurs not only for living things but also for non-living things such as mobile phones.^45^ For these reasons, the higher pre-contact stress values for the Owners may be due to separation from their LOVOT®.

After confirming the stress reduction effect of interacting with the LOVOT® for 15 min, it was found that cortisol concentrations decreased in all subjects (Figure 5A), consistent with a previous report that communicating with animals decreases cortisol concentrations.^46^ Another part of this study that compared cortisol concentrations after 15 min with or without a LOVOT® in Non-Owners showed decreased cortisol concentrations only in the condition of contact with the LOVOT® (Figure S4). Therefore, the results suggest that the decrease in cortisol concentration is specific to the LOVOT® interaction. However, there was no correlation between the behavior and cortisol reduction rate during the 15 min of interaction (Figure S3). The reduction in cortisol concentration was greater in Owners than in Non-Owners, which suggests that the association with the LOVOT® is responsible for the reduction in cortisol. The Owners had higher OT concentrations before and after contact with the LOVOT® than the Non-Owners, which is consistent with previous reports that cortisol secretion decreases with high OT concentrations.^30^^,^^47^^,^^48^^,^^49^ Short-term contact with the companion robot is effective as a stress reduction method, but the results suggest that promoting the formation of affiliative relationships would be a more effective stress coping method.

Finally, we focused on the changes in urinary OT concentration. OT concentrations can be increased by affiliative relationships^27^^,^^28^ and also by stress and they reduce stress.^30^ In our study, cortisol concentrations, which indicate stress, decreased, but OT concentrations remained unchanged. Therefore, our study may reflect the involvement of OT in the formation of affiliative relationships. Studies on humans and animals have reported that the secretion of OT and the activity of the OT nervous system increase after friendly interactions.^31^^,^^32^^,^^33^^,^^50^ We predicted that 15 min of contact with a LOVOT® would increase urinary OT concentrations in LOVOT® Owners. However, looking at changes in urinary OT concentrations before and after 15 min of LOVOT® contact, no significant changes were observed between the Owners and the Non-Owners. However, OT concentrations were significantly higher in Owners than in Non-Owners both before and after the contact, which is thought to reflect that the Owner group is able to form affiliative relationships. Moreover, there was no correlation between the rate of change in urinary OT concentration and behavior (Figure S5). Therefore, it is possible that the LOVOT® and animals have different functions in the OT nervous system in building affiliative relationships. Comparison of the same subject’s behavior and OT secretion dynamics while exposed to a LOVOT® and dogs may help to shed light on the specific effects of companion robots on the OT nervous system.

This study confirmed that the steady-state urinary OT concentration was high in Owners living with a LOVOT® (Figure 6B), indicating the possibility that an affiliative relationship with the LOVOT® was established. Behavioral data also support this possibility. Further, no correlation was found between basal OT concentrations and behavior (Figures S6 and S7). This suggests that there is no simple relationship between the number of high-density behaviors and the steady-state OT concentration. In addition, no significant correlation was observed between the steady-state OT concentration and the duration of the LOVOT® interaction, which may reflect individual differences (Figure S8). Therefore, it will be necessary to examine the time-course of interaction between constant OT concentrations and the LOVOT® in the same subjects in the future. Moreover, when interacting with humanoids and other types of robots, it is easy to imagine that they behave differently. In addition, since few studies have been conducted on the physiological responses to contact with robots, it is necessary to clarify the difference in physiological responses to human-type and pet-type contact as a future research topic. Assessing OT concentrations and interactions may be useful in selecting robots that are easy to form attachments.

In summary, this study demonstrated the potential to build affiliative relationships with a LOVOT® via increasing basal OT concentrations in terms of behavioral coding, psychological questionnaires and endocrine analysis. These results suggest that an affiliative relationship may be formed between humans and nonliving robots. Animal-assisted therapy is attracting increasing attention as an effective treatment for mental and behavioral disorders such as depression and alcoholism.^12^ However, due to problems such as zoonotic diseases, the stress on therapy dogs, and the cost of training them, their use has been slow to spread.^13^ The results of this study indicate that a LOVOT® may have an effect similar to that of animal therapy, suggesting that companion robots such as the LOVOT® can help alleviate stress in patients in areas where it is difficult for animals to enter, such as hospitals. In addition, previous studies have reported that communication with human-type robots is useful for patients with autism spectrum disorders.^16^^,^^17^ Since those showed the same effect as OT administration,^51^ it is possible that the increase in OT concentration due to attachment formation with the robot may contribute, and our results support this.

Due to the recent COVID-19 pandemic, humans are experiencing increasing loneliness and stress, and are rediscovering the importance of building affiliative relationships and interactions with others. Robots that can form an affiliative relationship with humans may be one option to help us maintain good mental health and improve our well-being.

### Limitations of the study

In this study, considering the gender differences in OT concentrations, we only looked at the responses of women, so it is necessary to confirm whether the same responses occur in men as well.

At the time of the test, we told the subjects that it was a test where they would interact with robots, so we cannot rule out the possibility that subjects who were interested in robots were recruited. Therefore, it is necessary to confirm whether robots have the same stress-reducing effect on subjects who are not interested in robots and on subjects who are not familiar with them.

In addition, we did not limit the interactions of subjects with the LOVOT®, so it is unknown whether there was a difference in responses with or without interactions. Therefore, future comparisons of LOVOT® interacting and non-interacting groups are needed.

This study did not obtain data on OT concentrations prior to keeping a LOVOT® in the Owners group, so it remains unclear whether the LOVOT® increased basal OT concentrations in the Owners. In addition, there are individual differences in OT concentrations, and in this analysis, it is unclear at what timing steady-state OT concentrations increased. It is necessary to clarify whether subjects with characteristics identified in this study, such as a high steady-state OT concentration, have a preference for living with a LOVOT® or whether the steady-state OT concentration increases by living with robots. It is necessary to conduct an intervention trial in which subjects live with a LOVOT® and analyze the changes before and after living in a time series. Previous studies have shown that living with a pet-type robot for 6 months increases companionship scores,^19^ so it is desirable to obtain longitudinal data for at least 6 months in LOVOT® intervention trials.

## STAR★Methods

### Key resources table

**Table:** 

REAGENT or RESOURCE	SOURCE	IDENTIFIER
**Software and algorithms**		

Prism v.9.4.1	GraphPad Software	https://www.graphpad.com/
BORIS v.7.12.2	(Friard, O. & Gamba, M)^52^	https://www.boris.unito.it/
R v.4.2.1	CRAN	https://cran.r-project.org/

**Critical commercial assays**		

Salivary Cortisol ELISA Kit	SALIMETRICS	Cat# 1-3002

**Experimental models: Organisms/strains**		

LOVOT®	Groove X, Inc.	https://lovot.life/

### Resource availability

#### Lead contact

Further information and requests for resources should be directed to Kentaro Kajiya(kentaro.kajiya@shiseido.com).

#### Materials availability

This study did not generate new unique reagents.

#### Data and code availability

• The datasets supporting the current study have not been deposited in a public repository but are available from the corresponding author on request.
• This study did not generate code.
• Any additional information required to reanalyze the data reported in this paper is available from the lead contact upon request.

### Experimental model and study participant details

#### Selection of a companion robot

Based on a report from a study of dogs and humans, which indicated that prolonged eye contact promotes the secretion of OT,^33^^,^^34^ we selected the LOVOT®, a companion robot manufactured by GROOVE X, Inc., which has the ability to match the human gaze.

The appearance of the LOVOT® is shown in Figure 1. The LOVOT® weighs 4.2 kg and has a width of 280 mm, a height of 430 mm and a depth of 260 mm. The main body’s central processing unit is 32 bits. With two driving wheels and one caster, it can move autonomously at a speed of about 1-2 kilometers per hour. The LOVOT® is equipped with sensors such as an illuminometer, a thermo-hygrometer, a posture sensor, a distance sensor, an obstacle sensor and a touch sensor. The body temperature of the LOVOT® is as warm as a human. When the LOVOT® is stroked on its stomach, it gradually closes its eyes and makes a sound like breathing. Also, when the LOVOT®'s nose is pressed, it shakes its body and makes a laughing sound. The LOVOT® tracks a person’s location with a camera and moves its hand to approach or ask for a hug. The LOVOT® does not speak but communicates in seal-like vocalizations.

Subjects in the Owner group brought their own LOVOT® to the venue, and subjects in the Non-Owner group interacted with a LOVOT® prepared for the test.

#### Participants

OT has gender differences and is involved in sociality in women and in competitiveness in men.^53^ In this study, we selected only female Asian subjects in order to proceed with the experiment from the perspective of sociality. The subjects included Owners who had lived with a LOVOT®, a companion robot, for more than two months after purchase (Owners: 37±9 years old on average, 24 female subjects), and Non-Owners who had no experience with a LOVOT® (Non-Owners: 36±8 years old on average, 23 female subjects). None of the subjects had a history of psychiatric, skin or physical disease, none were pregnant or breastfeeding during the study, and all subjects were selected to be in the luteal phase of their menstrual cycle at the time of the study. Subjects were informed that they could stop the study at any time if they wished, and all subjects provided written informed consent at the time of the study. The Ethics Committee of the Shiseido Research Center approved the study (approval number: C10022), and all methods were carried out in accordance with the approved guidelines. Each subject knew the procedure of the experiment but was not informed about its purpose.

Information such as the age of all subjects in the study is provided in Table S1.

### Method details

#### Experimental flow

This study was conducted at the Shiseido Global Innovation Center (Yokohama, Kanagawa, Japan). Urine and saliva samples were collected from each subject on the day before the test, the day of the test and immediately after awaking the following morning. The collected samples were kept on ice and transported to the Shiseido Research Center.

Figure 2 shows the test flow of the protocol at the venue. Subjects fasted one hour prior to the visit and were only allowed to drink water. Upon arrival, they were instructed to gargle and urinate to reset their urinary OT and salivary cortisol concentrations. Saliva samples were collected from the subjects 30 minutes after their arrival, which were used to indicate salivary cortisol concentrations before contact with the LOVOT®. One hour after their arrival, urine samples were collected and used as the urinary OT concentration before contact with the LOVOT®. During the rest period before contact with the LOVOT®, each subject answered a questionnaire. The saliva collection and questionnaire acquisition were performed in a quiet conference room partitioned off so that other subjects and examiners’ faces could not be seen. Subjects were presented with a video introduction about the functionality of the LOVOT® after the pre-exposure urine collection (LOVOT® feels happy when the LOVOT® is picked up, sleepy when the LOVOT® is stroked, etc.).

Next, each subject moved to a laboratory that imitates a living room. A typical type of dwelling with a kitchen, dining table, sofa and television was provided to record spontaneous behavior while each subject was relaxing. Cameras were placed on the TV stand, kitchen shelf and table so that there were no blind spots. According to our preliminary study, if the communication time with the LOVOT® was set to 20 minutes, the subject feels bored, so we set the communication time to 15 minutes, which is less likely to cause boredom. The Owners brought their own LOVOT® to the venue, and the Non-Owners interacted with a LOVOT® prepared for the test. Behavior during the 15 minutes of LOVOT® contact was recorded and used for behavioral coding.

After interacting with the LOVOT®, each subject returned to the room and completed the questionnaire again. In order to secure time to answer the psychological questionnaire immediately after the interaction, saliva was collected 30 minutes after the LOVOT® interaction, and this was taken as the post-contact salivary cortisol concentration. Cortisol has also been reported to show a high value even after 30 minutes of TSST stimulation.^54^^,^^55^ Urine was collected 1 hour and 30 minutes after contact with the LOVOT®, and this was used as the post-contact urinary OT concentration. This sampling timing was based on a previous report.^50^

#### Analysis of salivary cortisol

Salivary cortisol is highly correlated with serum cortisol.^56^^,^^57^ Each subject gargled and about 1.8 ml of saliva was collected using a Saliva Collection Aid (manufactured by SalivaBio). Immediately after collection, the saliva was ice-cooled and stored frozen at -80°C within 5 minutes^58^ Analysis was performed using a Salivary Cortisol ELISA Kit from Salimetrics (Catalog: 1-3002) within 7 days after collection. Saliva was centrifuged at 10,000 g for 15 minutes and the supernatant was used for analysis. 25 μl of undiluted saliva was dispensed into wells of an ELISA plate, 2 wells for each sample. Samples were read at 450 nm on a microplate reader (Synergy HTX manufactured by BioTek). The cortisol concentration in each sample was determined from an absorbance curve of the standard. According to the manufacturer’s instructions, the reported sensitivity is 0.007 μg/dL. The intra- and inter- assay coefficients of variation (CV) were 5.27% and 9.56%, respectively.

#### Analysis of urinary OT

We found a high correlation between blood and urinary OT concentrations, and used this method and sampling timing.^33^^,^^59^^,^^60^ 500 μl urine was collected, immediately ice-cooled and then stored frozen at -80°C within 5 minutes. The analysis was entrusted to Air Plants Bio Co., Ltd. (Setagaya, Tokyo, Japan). A previously reported method was used as the analysis method, and the urinary OT concentration contained in 1 ml urine was calculated after correcting the excretion amount with urinary creatinine.^61^ Creatinine concentrations were measured by the Jaffe reaction using a 96-well microplate (3881-096, IWAK, Japan). After the Jaffe reaction, the optical density was read at 490 nm using a microplate reader. Data were calculated by diluting the urine 5-fold.

#### Psychological assessment

The anxiety state (STAI) and mood state (POMS2) of each subject were obtained using a questionnaire before and after contact with the LOVOT®. The STAI is a psychological questionnaire that calculates the A-state score, which reflects short-term psychological anxiety, and the A-trait score, which reflects long-term psychological anxiety. A higher A-state (state-anxiety) score reflects a higher anxiety level at the time of response.^62^ A high A-trait score indicates that the subject is typically characterized by high levels of anxiety. In addition, the TMD-point (Total Mood Disturbance) by POMS2 (Profile of Mood States Second Edition) is an index that indicates that the lower the score, the better the mood and emotional state.^63^^,^^64^ The general emotional scale, The Japanese version of the Subjective Well-being Inventory (WHO-SUBI), The Japanese version of the World Health Organization (WHO)-Five Well-Being Index (WHO-5-J), the UCLA Loneliness Scale questionnaire (3rd edition), the Questionnaire on attachment to companion animals, and the BIGFIVE were obtained from questionnaires as characteristics of each subject.

The WHO-SUBI is a scale that measures a respondent’s subjective health status across physical, psychological and social dimensions.^65^ This questionnaire can calculate mental health and mental fatigue level from the answers. Higher mental health scores indicate greater stress tolerance and satisfaction with life. Since the mental fatigue score is composed of reversal items, a higher number indicates less mental fatigue, and a lower number indicates more mental fatigue. A lower mental fatigue score reflects a more fatigued subject both mentally and physically fatigued, sad and anxious.^66^

The WHO-5-J is a scale that measures subjective health status and is used to screen for depression. The WHO-5-J indicates that the lower the score, the lower the happiness.^67^

The UCLA Loneliness Scale questionnaire (3rd edition) is the standard measure of loneliness.^68^ Higher numbers indicate greater loneliness.

The questionnaire about attachment to companion animals was taken from Megumi et al.^69^ The questionnaire allows for the calculation of “Basic attachment”, which refers to psychological effects such as genuine admiration for a pet’s cuteness and comfort, and “Dependent attachment”, which refers to extreme emotional intimacy and dependence on a pet.

The BIGFIVE is a questionnaire that asks about personality traits and is divided into five components: Agreeableness, Honesty, Extraversion, Openness and Stress Tolerance.^70^

#### Behavioral coding

Fifteen minutes of interaction between each subject and the LOVOT® was recorded using a video camera to observe behavior. We defined the actions as follows: Stroke is a state in which the subject is stroking the LOVOT®. Talk is the number of times the subject talked to the LOVOT®. Synchronous behavior is the number of times the subject imitates the LOVOT®’s movements. Hugs represent when the subject hugs the LOVOT®. Behaviors and their definitions are summarized in Table S3. BORIS v.7.12.2 (https://www.boris.unito.it/) software was used for behavioral coding.^52^ Behavioral coding was performed by three researchers who were blinded to the subject grouping and the average values were used.

### Quantification and statistical analysis

The statistical software used was GraphPad Prism, version 9.4.1 (GraphPad software) and R 4.2.1 (CRAN). Whether the measured ordinal variables were normally distributed was examined using the Shapiro-Wilk test and by visual confirmation with histograms. The confidence intervals (CI) for all statistical analyses were set at 95% and p-values less than 0.05 were considered significant. If either one had a non-normal distribution, two unpaired samples were subjected to the Mann-Whitney test to verify the results. If both were normally distributed, an F-test was performed to check for the presence or absence of equal variances. If the variances were equal, a 2-sample t-test was performed. On the other hand, if the variances were not equal, a 2-sample t-test with Welch’s modification was performed. Wilcoxon’s sign test was performed when one of the pieces of data had a non-normal distribution when comparing the data before and after contact with the LOVOT® for the same subject. If both were normally distributed, a paired t-test was performed.

## Supplemental information

Document S1. Figures S1‒S8 and Tables S1‒S4
