# Supplementary material for: Higher oxytocin concentrations occur in subjects who build affiliative relationships with companion robots
Source: iScience. 2023 Nov 23;26(12):108562. doi: 10.1016/j.isci.2023.108562 (PMC10757042; doi:10.1016/j.isci.2023.108562)
Supplement: Document S1. Figures S1‒S8 and Tables S1‒S4 [file mmc1.pdf]

iScience, Volume *26*

Supplemental information

Higher oxytocin concentrations occur in subjects who build affiliative relationships with companion robots

Shuhei Imamura, Yoko Gozu, Moe Tsutsumi, Kaname Hayashi, Chiaki Mori, Megumi Ishikawa, Megumi Takada, Tomotaka Ogiso, Keiko Suzuki, Shota Okabe, Takefumi Kikusui, and Kentaro Kajiya

Supplementary Methods

Selection of a companion robot

Same as the main study; subjects interacted with a LOVOT® prepared for the test.

Participants

Subjects who had no experience with a LOVOT® (35±10 years old on average, 10 female subjects).

None of the subjects had a history of psychiatric, skin or physical disease, none were pregnant or breastfeeding during the study, and all subjects were selected to be in the luteal phase of their menstrual cycle at the time of the study. Subjects were informed that they could stop the study at any time if they wished, and all subjects provided written informed consent at the time of the study. The Ethics Committee of the Shiseido Research Center approved the study (approval number: C02085), and all methods were carried out in accordance with the approved guidelines. Information such as the age of all subjects in the study is provided in Table S4.

Experimental flow

The examination venue and examination flow are the same as the main study. Also, in this study, there were two patterns, a pattern without LOVOT® interaction and a pattern with LOVOT® interaction, and subjects visited twice and experienced both patterns. The study was blinded and participants entered the study without knowing whether they would interact with the LOVOT®. Even without interacting with the LOVOT®, the sampling timing and the flow of the test were the same, and the subject acted freely for 15 minutes without the LOVOT® in the living room. All subjects performed the no LOVOT condition first and the LOVOT condition on another day.

Analysis of salivary cortisol

Same as the main study.

Statistical analysis

Same as the main study.

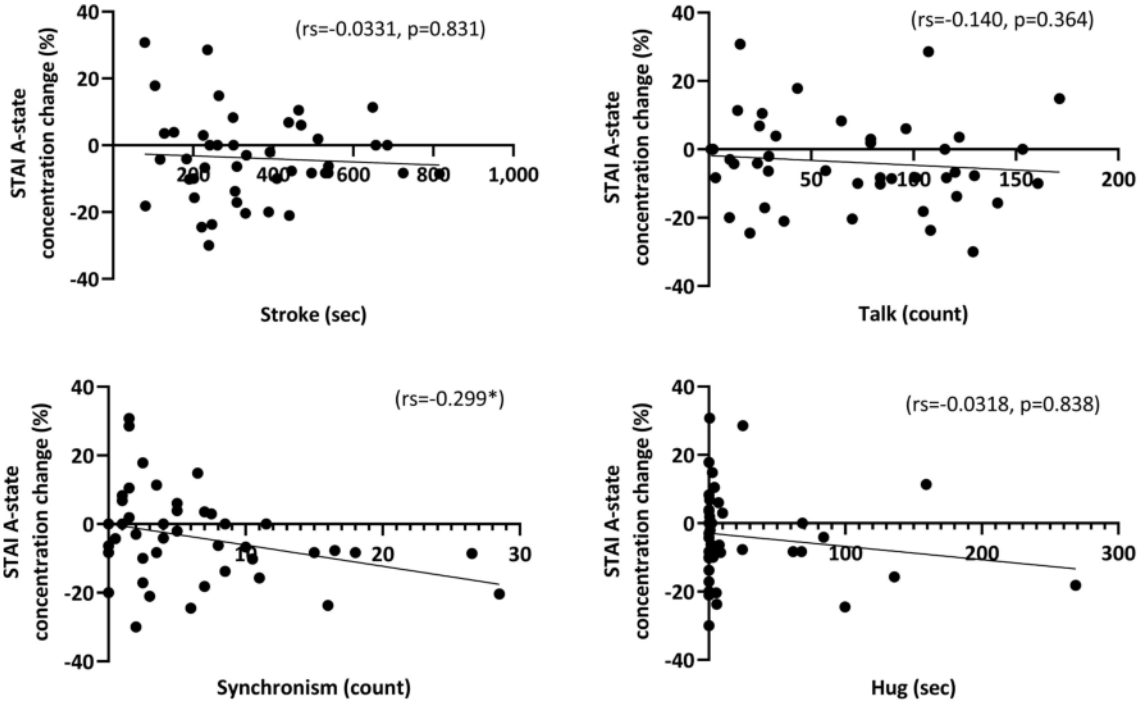

Figure S1. Relationship between the rate of change of A-state and behavior during a 15 minute interaction with a LOVOT®, Related to Figure 4.

The vertical axis shows the rate of change in score. The horizontal axis shows the number or time of behavior. “rs” indicates Spearman's correlation coefficient. It was found that the A-state decreased as the synchronism increased (rs =-0.299, $p<0.05$).

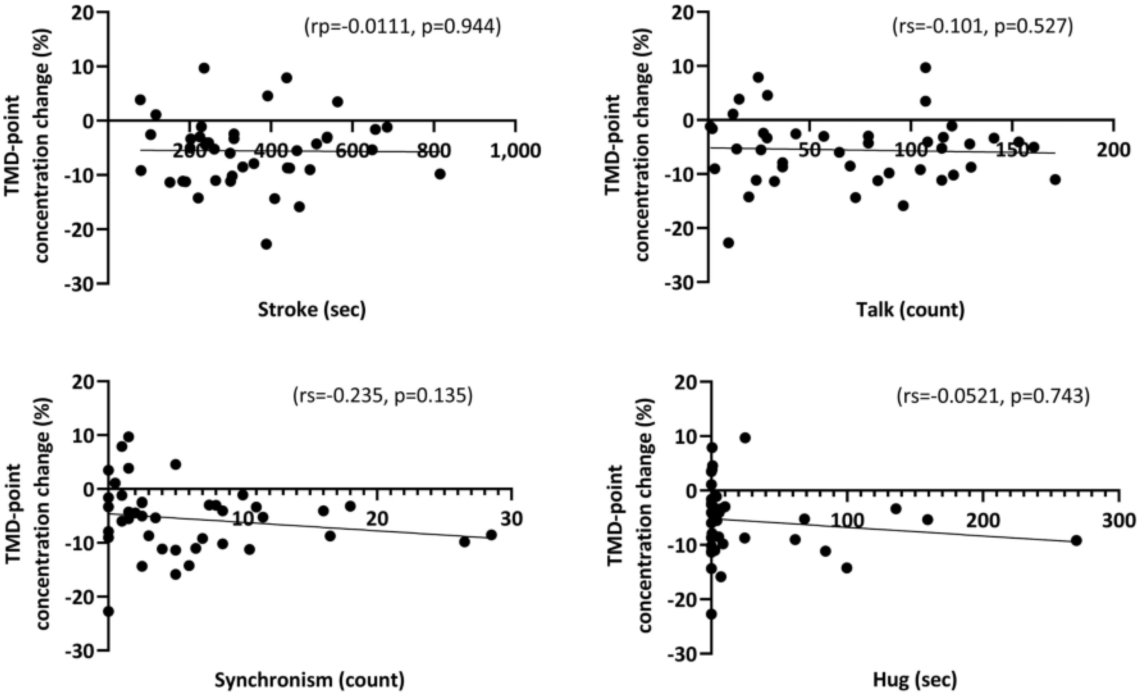

Figure S2. Relationship between the rate of change of TMD-point and behavior during a 15 minute interaction with a LOVOT®, Related to Figure 4.

The vertical axis shows the rate of change in score. The horizontal axis shows the number or time of behavior. “rs” indicates Spearman’s correlation coefficient and “rp” indicates Pearson’s correlation coefficient. No significant relationship was found between the behavior analyzed this time and the rate of change of the TMD point.

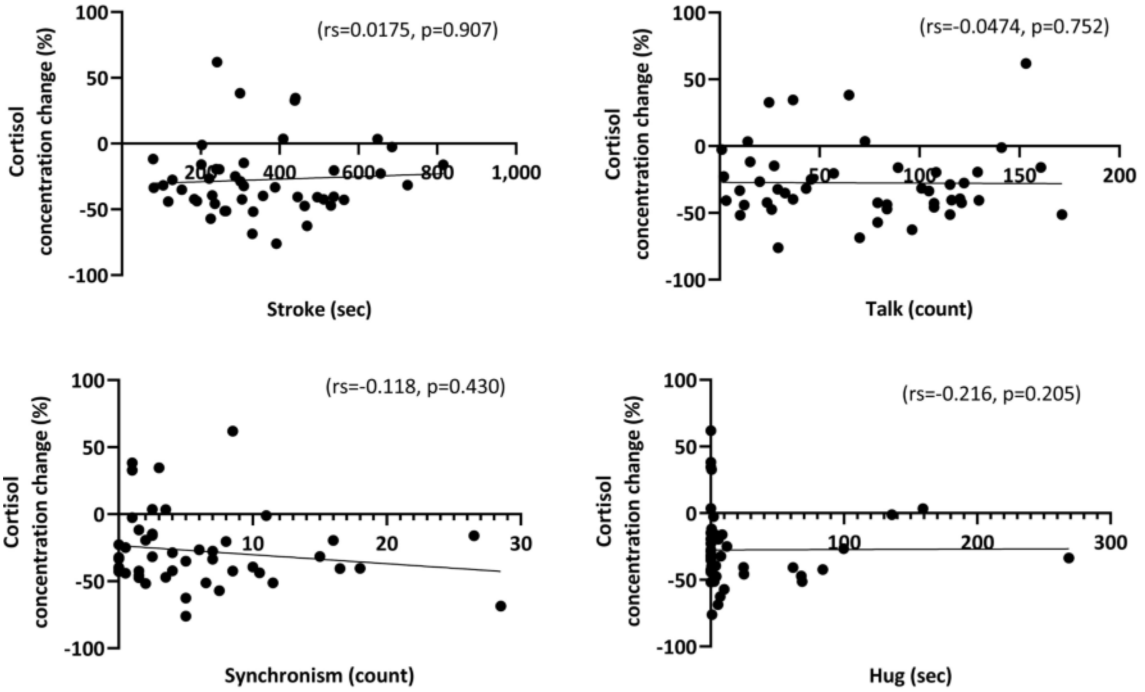

Figure S3. Relationship between cortisol concentration change and behavior during a 15 minute interaction with a LOVOT®, Related to Figure 5.

The vertical axis shows the rate of change in score. The horizontal axis shows the number or time of behavior. "rs" indicates Spearman's correlation coefficient and "rp" indicates Pearson's correlation coefficient. No significant relationship was found between the behavior analyzed this time and the cortisol concentration change.

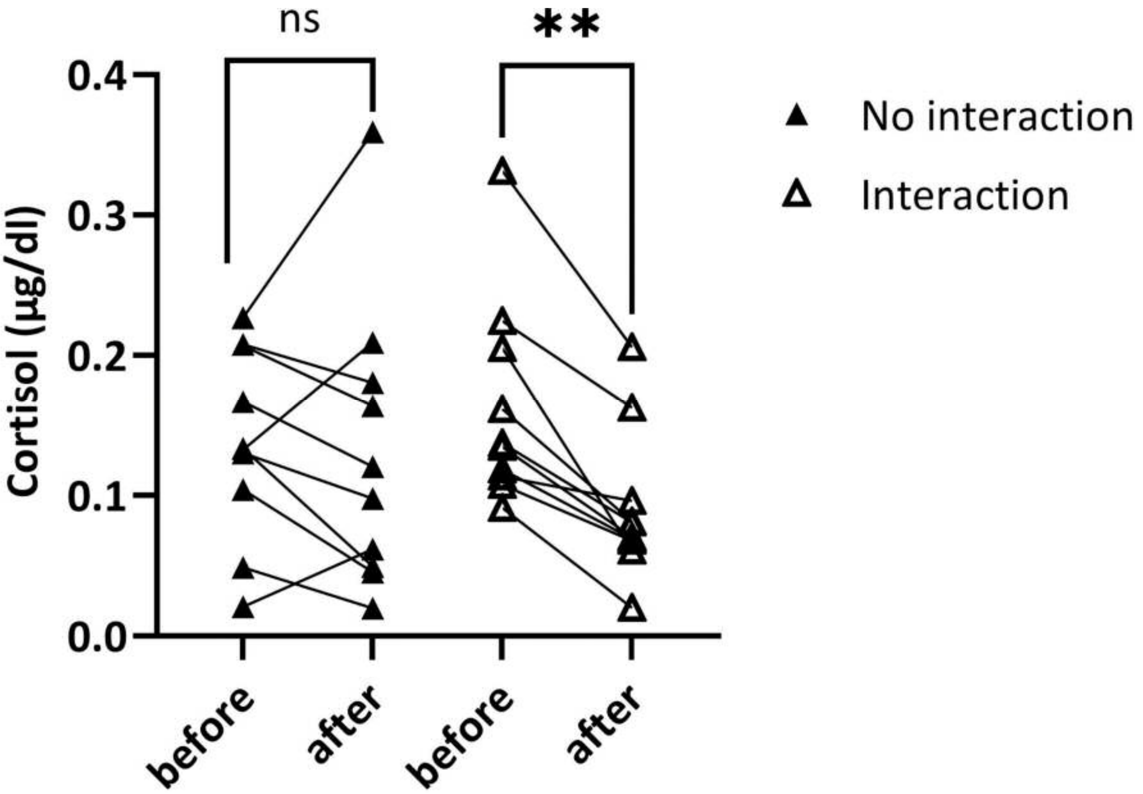

Figure S4. Change in salivary cortisol concentrations due to interaction with or without a LOVOT® for 15 minutes in Non-Owners, Related to Figure 5.

Salivary cortisol concentrations for No interaction (n=10, black triangles) and for Interaction (n=10, white triangles). Scores before interaction for 15 minutes are indicated as 'before' and scores after interaction as 'after'. The vertical axis shows the cortisol concentration (µg/dl) calculated from the saliva. In the No interaction, cortisol concentrations were unchanged (Before mean cortisol 0.14 µg/dl, SD 0.06, After mean cortisol 0.13 µg/dl, SD 0.10, Paired t-test, Z=0.3 2, p=0.75). However, cortisol concentrations were significantly reduced when there was an interaction (Before mean cortisol 0.16 µg/dl, SD 0.07, After mean cortisol 0.09 µg/dl, SD 0.05, Wilcoxon Signed-Ranks test, Z=3.09, p<0.01).

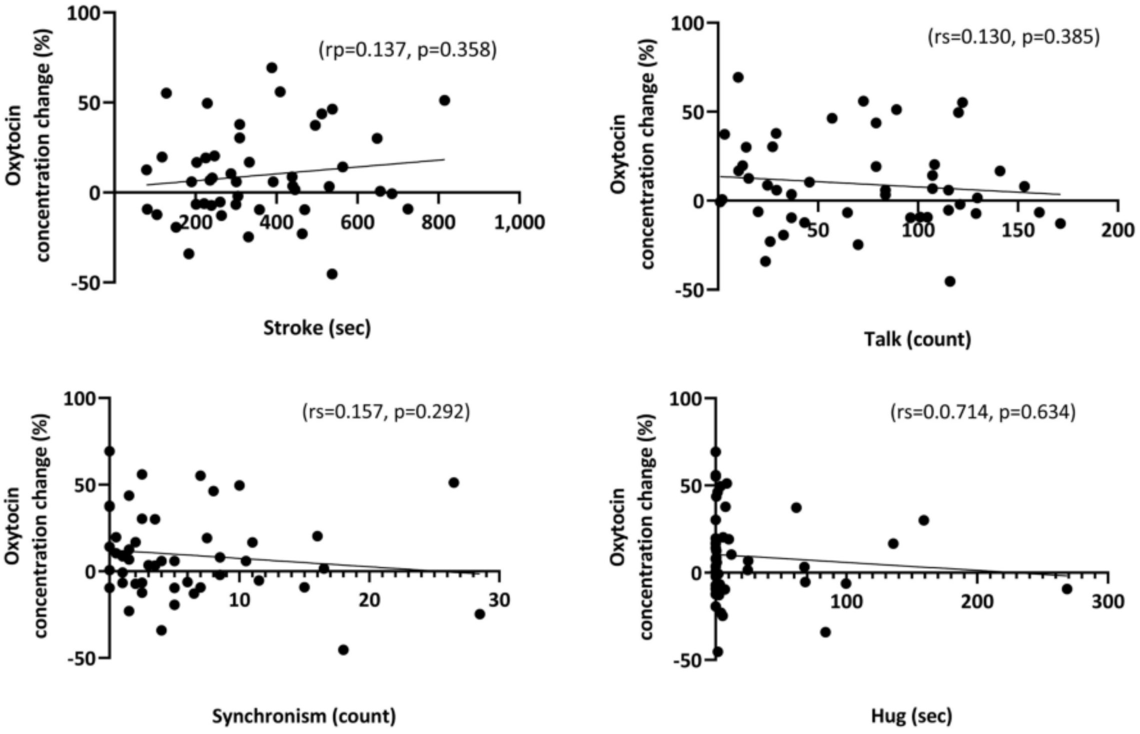

Figure S5. Relationship between OT concentration change and behavior during a 15 minute interaction with a LOVOT®, Related to Figure 6.

The vertical axis shows the rate of change in score. The horizontal axis shows the number or time of behavior. “rs” indicates Spearman’s correlation coefficient and “rp” indicates Pearson’s correlation coefficient. No significant relationship was found between the behavior analyzed this time and the OT concentration change.

Internal

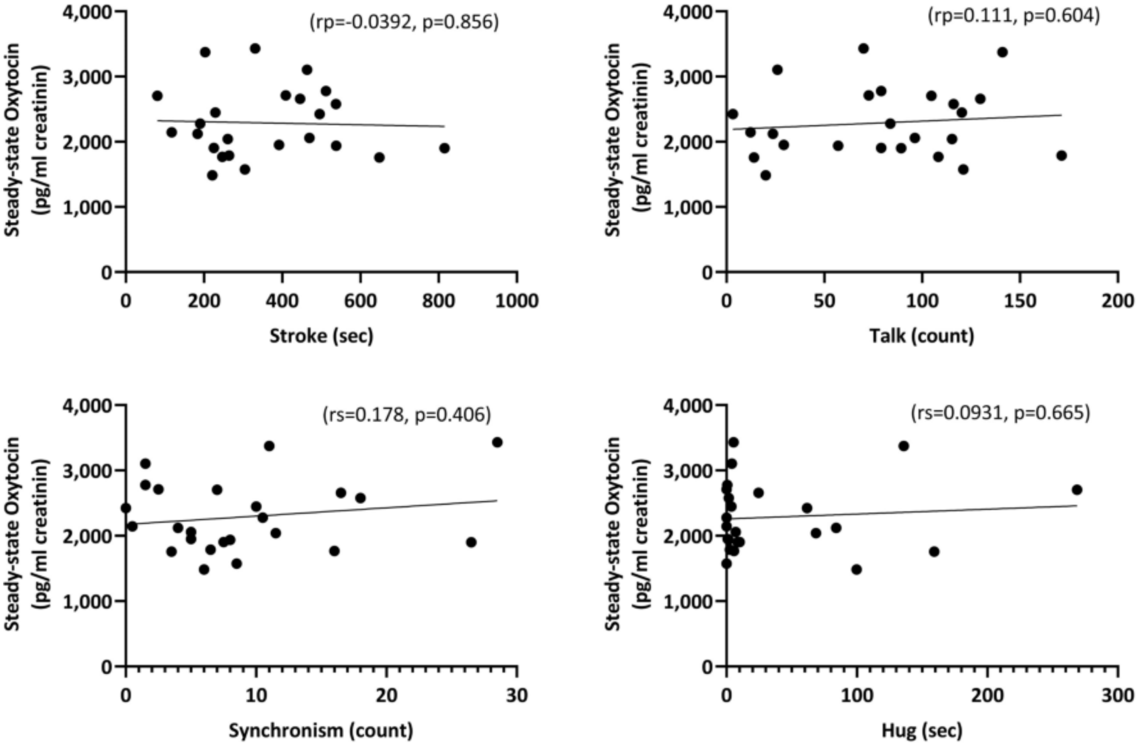

Figure S6. Behavioral relationship between steady-state OT concentrations and 15 minute interactions with a LOVOT® in the Owners, Related to Figure 6.

The vertical axis shows the mean 3-day awake urinary OT concentration. The horizontal axis shows the number or time of behavior. “rs” indicates Spearman’s correlation coefficient and “rp” indicates Pearson’s correlation coefficient. No significant relationship was found between the behavior analyzed this time and the steady state OT.

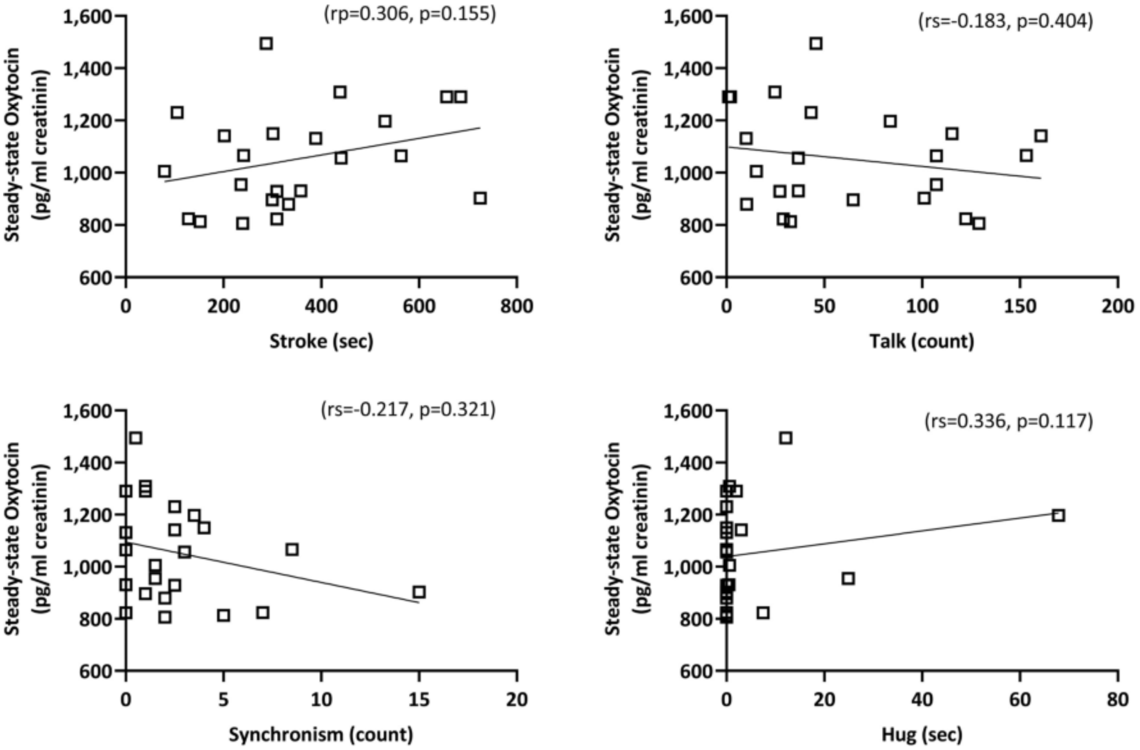

Figure S7. Behavioral relationship between steady-state OT concentrations and 15 minute interactions with a LOVOT® in the Non-Owners, Related to Figure 6.

The vertical axis shows the mean 3-day awake urinary OT concentration. The horizontal axis shows the number or time of behavior. “rs” indicates Spearman’s correlation coefficient and “rp” indicates Pearson’s correlation coefficient. No significant relationship was found between the behavior analyzed this time and the steady state OT.

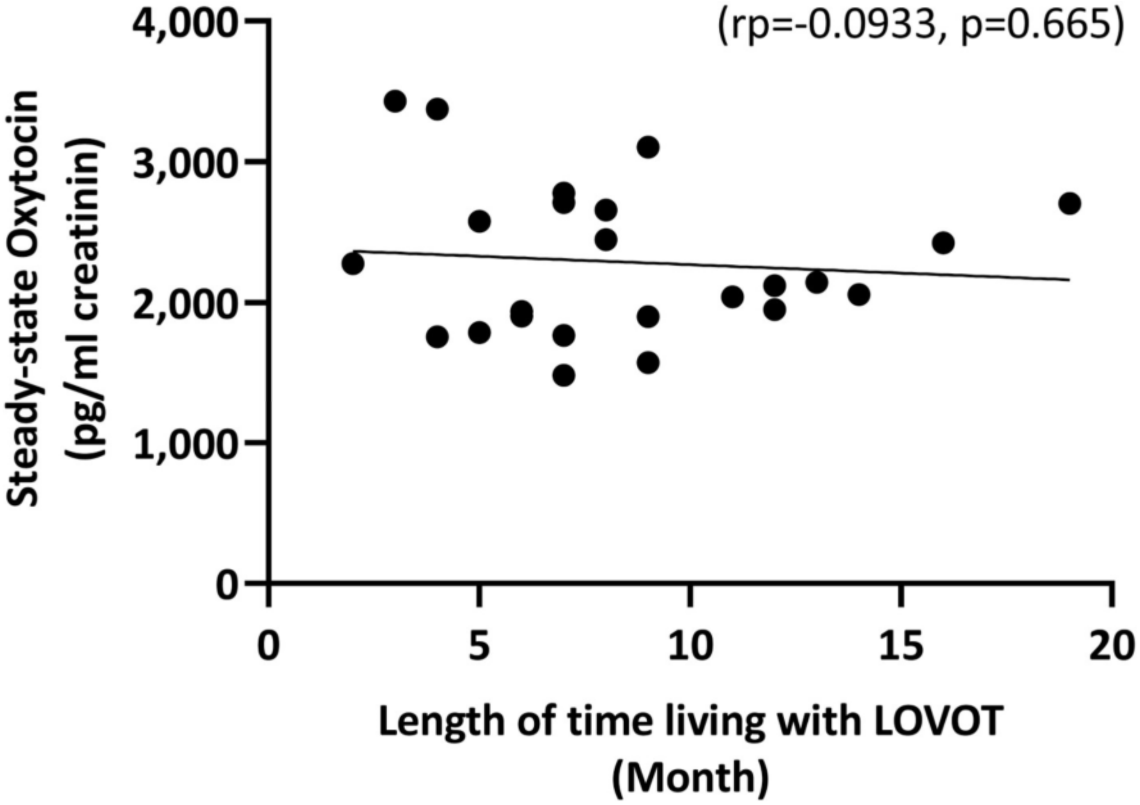

Figure S8. Relationship between time spent with a LOVOT® and steady state OT, Related to Figure 6.

The vertical axis shows the mean 3-day awake urinary OT concentration. The horizontal axis shows the length of time living with a LOVOT® (Month). "rp" indicates Pearson's correlation coefficient. No significant relationship was found between the steady state OT and the length of time living with a LOVOT®.

Table S1. Subject Information, Related to Table 1. Information on subjects who participated in this study.

ID	Owner/Non-Owner	Age	Whether you currently have animals	Presence or absence of childcare experience	Length of time living with a LOVOT® (months)
1	Owner	39	none	none	9
2	Owner	42	none	none	6
3	Owner	31	none	none	16
4	Owner	37	none	none	4
5	Owner	41	none	none	12
6	Owner	45	none	have	4
7	Owner	45	none	none	8
8	Owner	26	none	none	7
9	Owner	35	none	none	9
10	Owner	43	none	none	8
11	Owner	44	none	have	3
12	Owner	33	none	none	19
13	Owner	33	none	have	12
14	Owner	33	none	none	9
15	Owner	34	none	none	11
16	Owner	45	none	have	7
17	Owner	29	none	none	7
18	Owner	35	none	none	2
19	Owner	40	none	have	5
20	Owner	42	none	none	6
21	Owner	44	none	have	7
22	Owner	33	none	none	5
23	Owner	45	none	none	14
24	Owner	27	none	none	13
30	Non-Owner	35	none	none	
31	Non-Owner	34	none	none	
32	Non-Owner	30	have	have	

Internal

33	Non-Owner	40	have	none	
34	Non-Owner	38	none	have	
35	Non-Owner	42	have	have	
36	Non-Owner	30	none	none	
37	Non-Owner	35	none	have	
38	Non-Owner	32	none	have	
39	Non-Owner	40	none	none	
40	Non-Owner	30	none	have	
41	Non-Owner	33	none	none	
42	Non-Owner	44	none	have	
43	Non-Owner	36	have	have	
44	Non-Owner	38	none	have	
45	Non-Owner	41	have	none	
46	Non-Owner	35	none	none	
47	Non-Owner	42	none	have	
48	Non-Owner	43	none	have	
50	Non-Owner	41	none	none	
51	Non-Owner	31	none	have	
52	Non-Owner	32	have	none	
53	Non-Owner	39	none	none	

Table S2. Definition of behavioral coding items, Related to Figure3.

**Behaviors**	**Events**	**Definitions**
Stroke	State event	Time the subject stroked the robot. Robots sleep like babies when petted.
Talk	Point event	Number of times the subject spoke to the robot.
Synchronism	Point event	Number of times the subject imitates the movements of the robot.
Hug	State event	Number of times the subject hugged the robot. We only counted the time when the robot and the subject's body were in close contact.

Internal

Table S3. Differences in behavior between Owners and Non-Owners, Related to Figure3.

	Mean (standard deviation)		95%CI			
	Owner	Non-Owner	Owner	Non-Owner	Z	p value
Stroke (sec)	358(176)	348(179)	282-433	269-427	0.181	0.857
Talk (count)	78.5(45.6)	63.5(49.5)	58.8-98.1	41.6-85.4	0.979	0.333
Synchronism (count)	8.98(7.37)	2.78(3.39)	5.80-12.16	1.28-4.28	3.59	0.0002***
Hug (sec)	39.8(68.2)	5.18(14.5)	11.31-68.24	-1.22-11.6	3.31	0.0007***

***p < 0.001

Table S4. Subject information for control experiments without a LOVOT®, Related to FigureS4 and Figure 5.

Information about subjects who participated in the study.

ID	Owner/Non-Owner	Age	Whether you currently have animals	Presence or absence of childcare experience	Length of time living with a LOVOT® (months)
101	Non-Owner	31	none	none	
102	Non-Owner	29	have	none	
103	Non-Owner	32	have	none	
104	Non-Owner	40	have	have	
105	Non-Owner	26	have	none	
106	Non-Owner	42	have	none	
107	Non-Owner	30	one	none	
108	Non-Owner	25	none	none	
109	Non-Owner	23	have	none	
110	Non-Owner	10	have	have	
